# VIP treatment prevents embryo resorption by modulating efferocytosis and activation profile of maternal macrophages in the CBAxDBA resorption prone model

**DOI:** 10.1038/srep18633

**Published:** 2016-01-06

**Authors:** Lucila Gallino, Guillermina Calo, Vanesa Hauk, Laura Fraccaroli, Esteban Grasso, Mónica Vermeulen, Claudia Pérez Leirós, Rosanna Ramhorst

**Affiliations:** 1 Immunopharmacology Laboratory, School of Sciences, University of Buenos Aires. IQUIBICEN-CONICET; 2 Institute of Experimental Medicine IMEX-CONICET, National Academy of Sciences, Buenos Aires.

## Abstract

Successful embryo implantation occurs followed by a local pro-inflammatory response subsequently shifted toward a tolerogenic one. VIP (vasoactive intestinal peptide) has embryotrofic, anti-inflammatory and tolerogenic effects. In this sense, we investigated whether the *in vivo* treatment with VIP contributes to an immunosuppressant local microenvironment associated with an improved pregnancy outcome in the CBA/J × DBA/2 resorption prone model. Pregnancy induced the expression of VIP, VPAC1 and VPAC2 in the uterus from CBA/J × DBA/2 mating females on day 8.5 of gestation compared with non-pregnant mice. VIP treatment (2 nmol/mouse i.p.) on day 6.5 significantly increased the number of viable implantation sites and improved the asymmetric distribution of implanted embryos. This effect was accompanied by a decrease in RORγt and an increase in TGF-β and PPARγ expression at the implantation sites. Moreover, VIP modulated the maternal peritoneal macrophages efferocytosis ability, tested using latex beads-FITC or apoptotic thymocytes, displaying an increased frequency of IL-10-producer F4/80 cells while did not modulate TNF-α and IL-12 secretion. The present data suggest that VIP treatment increases the number of viable embryos associated with an increase in the efferocytic ability of maternal macrophages which is related to an immunosuppressant microenvironment.

From the perspective of the immune system, pregnancy involves the generation of a sterile inflammatory response that will be physiologically limited in its extent and duration by several immunoregulatory mechanisms^1^,^2^. This pro-inflammatory response is accompanied by tissue remodeling at the implantation site, associated with apoptosis processes that generate a cell turn over implicated in the maintenance of tissue homeostasis and allowing trophoblast invasion into maternal decidua^3^,^4^. Maternal immune system had to evolve mechanisms for tissue homeostasis maintenance in a tolerogenic microenvironment including the participation of specialized leukocyte subpopulations, such as regulatory T cells (Treg), uterine NK cells, decidual macrophages activated in an alternative profile and tolerogenic dendritic cells^5^,^6^,^7^ as well as various cytokines, chemokines, galectins and immune polypeptides^8^,^9^.

In this sense, the specialized Treg population (CD4 + CD25 + Foxp3+), essential for maternal tolerance of the conceptus, is stimulated through antigen-specific and antigen non-specific pathways thus exerting their suppressive actions in the critical peri-implantation phase of pregnancy^10^. This local tolerogenic microenvironment is also accompanied by a decrease in T-bet (transcriptor factor associated with a Th1 profile) and RORγt expression (transcription factor associated with a Th17 profile) in several *ex vivo* and mice models^11^,^12^,^13^,^14^,^15^. In humans, low frequency of Th17 cells was reported in the decidua in comparison to blood during early pregnancy^13^,^16^; and also a systemic increase in the ratio between Foxp3+ and IL-17-producing CD4+ T cells^13^.

Consistently with a tolerogenic microenvironment, maternal macrophages present a predominant alternative activation profile contributing to the production of IL-10 and TGFβ, wound healing mediators synthesis and the clearance of apoptotic bodies^5^,^17^. In this sense, the efferocytosis, or engulfment of apoptotic cells by macrophages, is an essential process that contributes to tissue homeostasis with a profound influence on inflammation resolution, through secretion of anti-inflammatory cytokines, such as TGF-β and IL-10^5^,^17^,^18^.

In this intricate network generated by maternal leukocytes at the maternal-placental interface, the role of the immune peptides is to allow the communication between immune and trophoblast cells^8^.

Particularly VIP, vasoactive intestinal peptide, mediates immune and nervous effector functions and has emerged as a potential effective treatment for inflammatory and autoimmune disorders based on its anti-inflammatory and tolerogenic effects^11^,^18^,^19^ as it was demonstrated in mouse models of inflammation through its action on macrophages and T cell VPAC receptors^20^,^21^,^22^.

In the feto-maternal context, VIP is present in viable implantation sites of normal mice where it induces an increase in Treg frequency^12^. However, lower levels of VIP and Foxp3 were found in implantation sites of prediabetic NOD (non-obese diabetic) mice, which are characterized by a Th1 systemic cytokine profile, correlating with a reduction in litter size and increased resorption rates^23^. In fact, pregnant NOD mice treated with VIP at gestational day 6.5, show a modulation of inflammatory signals at the early maternal-fetal interface and a partial improvement in the pregnancy outcome^12^.

Since the control of the initial inflammatory response after embryo implantation is crucial for a successful pregnancy outcome^1^,^2^ and considering that VIP mediates anti-inflammatory and tolerogenic immune effects, we hypothesized that VIP participates in homeostasis maintenance at the early maternal-placental interface inducing an immunosuppresant microenvironment through maternal macrophage efferocytosis associated with an alternative activation profile. The mating of CBA/J females (H2k) with DBA/2 males (H2d) provokes spontaneously high resorption rates associated with a failure in the maternal tolerogenic response, whereas mating CBA/J females with BALB/c mice, which also bear H2d antigens, ends in completely normal pregnancies offering the possibility to elucidate immunological mechanisms and modify them through different drug treatments^24^. In line with this, we propose that VIP treatment at the early post implantation window of pregnant CBA/J × DBA/2 mouse mating, would induce an immunosuppressant local microenvironment that could contribute to reach certain end-points of normal gestation, such as the number of viable embryo and a symmetric distribution along the uterine horns.

## Results

### VIP/VPAC system expression at the feto-maternal interface in CBA/J × DBA/2 matings: characterization in viable implantation sites

CBA/J × DBA/2 mating is a well-studied model of immunologically mediated peri-implantation pregnancy loss that shares features with human pregnancy complications. Embryos derived from mating CBA/J females with DBA/2 males showed an increased frequency of resorption rates compared with other strains or strain combinations^25^,^26^. Embryonic lethality in this mating is due to the rejection of the semiallogeneic placenta by several altered maternal immune mechanisms, such as a reduction in Treg frequency^24^.

**Figure 1:**
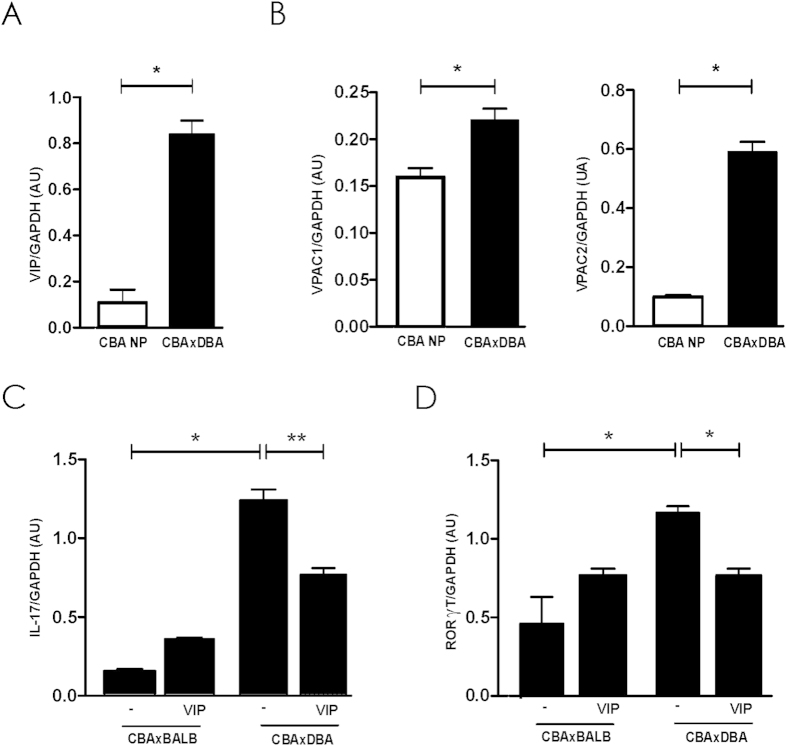
VIP/VPAC system at the feto-maternal interface in CBA/J × DBA/2 mating. Pregnant CBA/J ×DBA/2 females or CBA non pregnant mice were sacrificed at 8.5 day of gestation and implantation sites were obtained. (**A**) VIP and (**B**) VPAC receptors were evaluated by RT-PCR as described in Mat erial and Methods (**C**) Implantation site explants isolated from CBAxDBA or CBAxBALB/c matings were cultured in the absence/presence of VIP (100 nM) during 24 h and IL-17 and RORγt were evaluated by RT-PCR. Bands were semi-quantified with ImageJ® and intensity expressed in arbitrary units (AU) relative to GAPDH. Values represent mean ± S.E.M of at least 3 experiments (Mann Whitney test *p < 0.05).

First of all, we studied the expression of VIP and its high affinity receptors (VPAC_1_ and VPAC_2_) in viable implantation sites. As shown in Fig. 1A,B, pregnancy significanlty increase the expression of VIP and their receptors, VPAC_1_ and VPAC_2_. To assess if VPACs were functional, the implantation site explants were cultured in the absence/presence of VIP (100 nM) during 24 h and the expression of IL-17 and the transcription factor RORγt were evaluated by RT-PCR. As an additional control implantation sites from CBA/J females after mating with BALB/c male at the same gestational age were used. Figure 1C,D, show that the treatment with VIP *in vitro,* significantly reduced IL-17 and RORγt expression at the implantation sites. In fact, in those cultures performed in the absence of VIP, CBA/J × DBA/2 implantation sites displayed higher IL-17 and RORγt expression compared with those in the control mating cultured under the same conditions. The present results show that implantation sites of CBA/J × DBA/2 mating have a functional VIP/VPAC system and also suggest that they display an exacerbated Th17 profile in comparison with the control that can be modulated *in vitro* by VIP.

### *In vivo* VIP treatment increases the number of viable implantation sites and improves their asymmetric distribution

This pregnancy model with high resorption rates enables the evaluation of an immunomodulatory treatment and the study of different end points of pregnancy outcome as well as the isolation of implantation sites for further analysis. Therefore, CBA/J × DBA/2 female were treated *in vivo* with an intraperitoneal injection of VIP (2 nmol/mouse) or PBS (as control) in the early post-implantation period (at 6.5 day of gestation).

**Figure 2:**
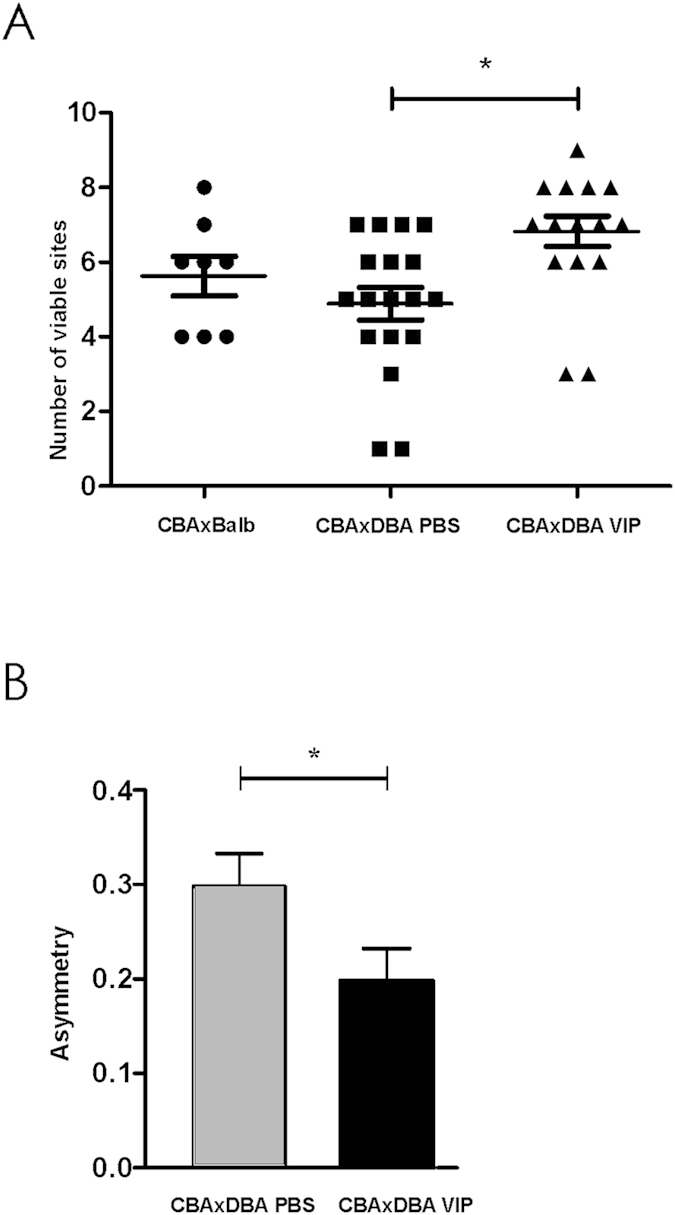
*In vivo* VIP treatment increases the number of viable implantation sites and improves the asymmetric distribution. CBA/J × DBA/2 females were treated *in vivo* with VIP (2 nmol/mouse) or PBS i.p. at day 6.5 of gestation. At d8.5 females were sacrificed and (**A**) number of viable implantation sites and (**B**) the asymmetric distribution of implanted embryos were evaluated as described in Material and Methods. Values are media ± S.E. of viable sites counted in 8 mice (control group), 18 mice (CBAxDBA, PBS group) and 15 mice (CBAxDBA, VIP group).

Figure 2A shows that VIP treatment significantly increased the number of viable implantation sites (day 8.5) in comparison with PBS-treatment, while did not modulate the number of sites with resorption. In addition, an improvement in the asymmetric distribution of implanted embryos was observed (Fig. 2B).

### VIP treatment decreases RORγt while increases tolerogenic mediators expression at the implantation sites

**Figure 3:**
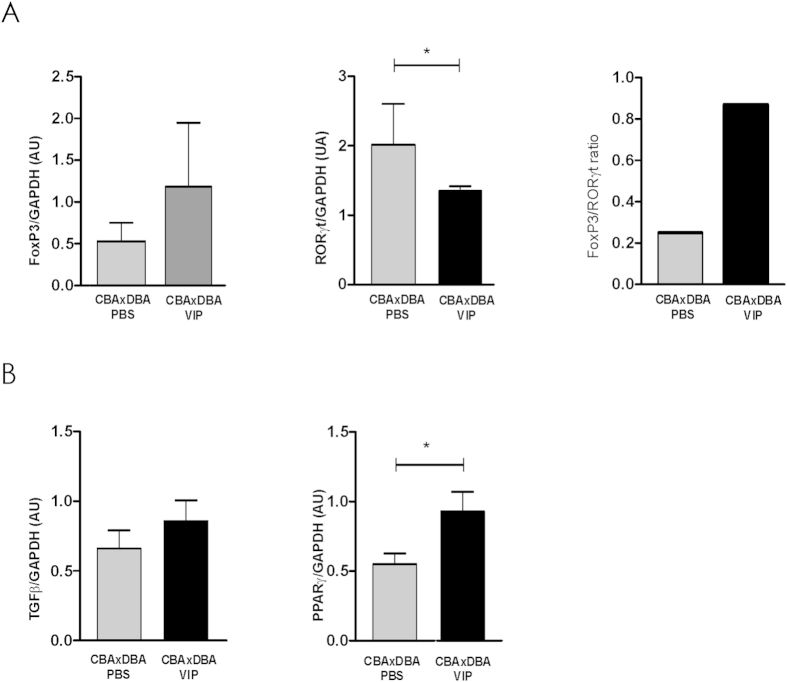
VIP treatment increases the Treg/Th17 ratio and the expression of tolerogenic mediators at the implantation sites. CBA/J × DBA/2 pregnant mice were treated *in vivo* with 2 nmol/mouse VIP or PBS i.p. at day 6.5 and the implantation sites were obtained at day 8.5. (**A**) Foxp3 and RORγt expression and (**B**) TGF-β and PPARγ expression were evaluated by RT-PCR. Bands were semi-quantified with ImageJ® and intensity expressed in arbitrary units (AU) relative to GAPDH. Values represent mean ± S.E.M of at least 3 experiments (Mann Whitney test *p < 0.05).

In line with the previous results, we analyzed whether VIP *in vivo* treatment could modulate the immune microenvironment at the implantation sites. CBA/J × DBA/2 pregnant mice were treated *in vivo* with VIP or PBS, as described, and at day 8.5 the implantation sites were obtained and Foxp3/RORγt expression ratio was evaluated by RT-PCR. Figure 3A shows that VIP treatment induced a significant decrease in RORγt expression, implying that the Treg/Th17 ratio is increased.

Moreover, we investigated TGF-β and PPARγ expression, on the knowledge that TGF-β is not only a pro-implantatory mediator but it is also associated with suppressor functions; and that PPARγ increased expression and/or activity is essential for the acquisition of alternative activation in macrophages^27^,^28^. Figure 3B shows that VIP treatment increased the expression of both tolerogenic markers, suggesting that VIP treatment induces a local tolerogenic microenvironment that would increase the number of viable embryos.

### VIP treatment increases maternal macrophage efferocytosis

The constitution of the maternal-placental interface involves apoptosis of trophoblast cells, smooth muscle and endothelial cells that, in turn, requires an immediate clearance of apoptotic cells by macrophages and their activation in an alternative profile (to prevent a deleterious inflammatory response)^5^,^29^. Hence, we first explored whether the treatment of CBA/J × DBA/2 pregnant mice with VIP could modulate the efferocytic ability of maternal macrophages using two complementary approaches: the efferocytosis of apoptotic thymocytes and of latex beads.

**Figure 4:**
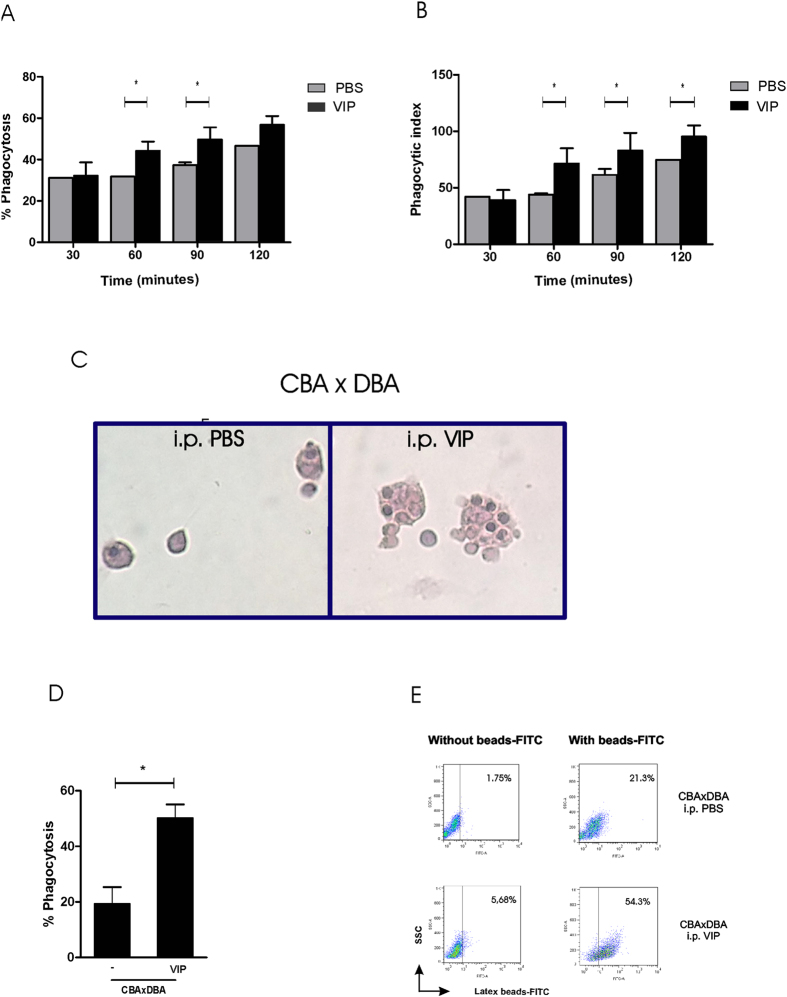
VIP treatment increases the maternal macrophage efferocytosis ability. Peritoneal macrophages from CBA/J × DBA/2 females were recovered at 8.5 day of gestation and cultured with syngeneic thymocytes induced to apoptosis in the absence or presence of VIP (10 nM). After, 30, 60, 90 and 120 min cells were stained with Hematoxilin–Eosin and the (**A**) % of efferocytosis and (**B**) the phagocytic index were analyzed by microscopy. (**C**) Representative images of macrophages recovered from pregnant CBA/J treated with PBS or VIP after apoptotic thymocytes engulfment. (**D**) Maternal peritoneal macrophages were recovered at day 8.5 after *in vivo* treatment with VIP or PBS and cultured with with latex-beads-FITC conjugated. After 90 min the percentage of efferocytosis was quantified by FACS analysis. (**E**) Representative dot plot profile previous and after the efferocytosis assay in absence or presence of VIP.

For the first approach, the efferocytosis of apoptotic thymocytes, maternal macrophages were recovered at day 8.5 after *in vivo* treatment with VIP, as previously described, and were co-cultured with syngeneic thymocytes induced to apoptosis. After, 30, 60, 90 and 120 min cells were stained with Hematoxilin–Eosin and analyzed by microscopy. As describes Fig. 4A, VIP treatment significantly increased the % of efferocytosis after 60 and 90 min of incubation with apoptotic thymocytes. This was accompained by a signficant increase in the phagocytic index (Fig. 4B). Figure 4C shows representative microphotographs of macrophages recovered from pregnant CBA/J treated with PBS or VIP after apoptotic thymocytes engulfment.

With the second approach, we could observe that the *in vitro* treatment with VIP increased the phagocytosis of latex beads by maternal macrophages, strongly suggesting that the increase in the efferocytosis observed in peritoneal macrophages was due to a direct effect of the peptide when injected to pregnant mice (Fig. 4D). Figure 4E shows a representative dot plot profile previous and after the efferocytosis assay in absence or presence of VIP.

### VIP treatment modulates macrophage cytokine production

Finally, we evaluated whether VIP treatment *in vivo* modulates the profile of cytokines produced by maternal macrophages from CBA/J pregnant mice. Therefore, after the efferocytosis assay, macrophages from pregnant CBA/J mice treated with PBS or VIP were washed and remained in culture for additional 24 h, then supernatants were collected for ELISA determinations and cells recovered for FACS analysis.

**Figure 5:**
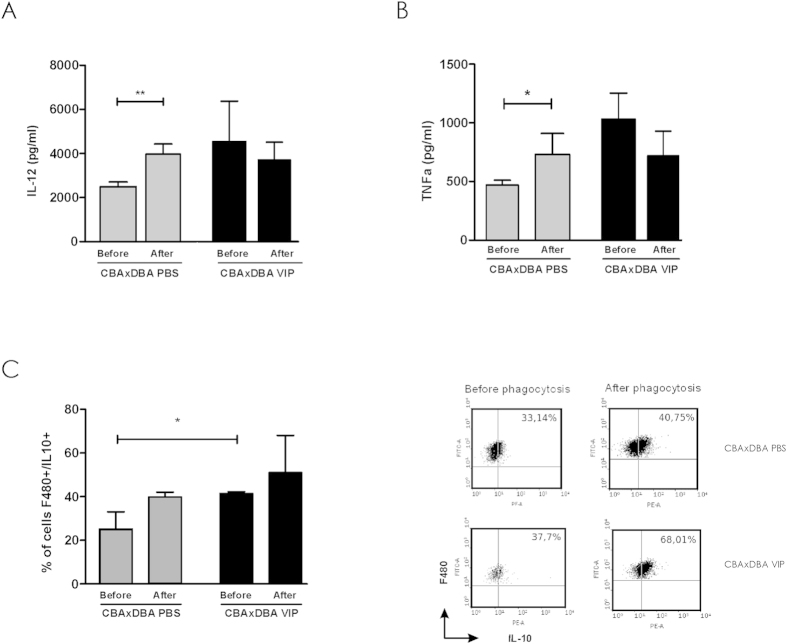
VIP treatment modulates macrophage cytokine production After the efferocytosis assay, macrophages from pregnant CBA/J mice treated with PBS or VIP (2 nmol/mouse) were washed and remained in culture for additional 24 h, then supernatants were collected and (A) IL-12 and (B) TNF-α were quantified by ELISA. (**C**) Cells were recovered and the frequency of F4/80 + IL-10+ analyzed by FACS. The left pannel shows representative dot plot with the frequency of of F4/80 + IL-10 from famele treated *in vivo* with PBS or VIP, previous and after the efferocytosis assay.

As shown in Fig. 5A, the control mice that were not treated with VIP had higher levels of IL-12 while the CBA/J group treated with VIP did not modulate IL-12 levels (Fig. 5A). The same pattern of secretion was observed for TNF-α (Fig. 5B). In contrast, VIP treatment increased the frequency of F4/80 + IL10+ cells even before the efferocytosis assay, suggesting that VIP treatment might modulate maternal macrophages *in vivo* (Fig. 5C).

## Discussion

Homeostasis maintenance at the feto-maternal interface is the result of multiple processes that occur at local and systemic levels which require several ongoing signals and checkpoints. Immune cells involved in the control of the early inflammatory response have a central role at the maternal-placental interface such as the activation of macrophages in an alternative profile or M2 instead of the classical inflammatory profile or M1^5^,^17^. In the present work, we analyzed the immunoregulatory role of VIP in the CBA/J × DBA/2 mating with high resorption rate associated with a failure in the tolerogenic maternal response. The present results provide new data on the *in vivo* treatment with VIP, which prevents embryo resorption and improves pregnancy end points evidenced as an increase in the number of viable embryo with a symmetric distribution in the uterine horns.

The ability of VIP to improve pregnancy outcome is based in its immunomodulatory effects observed not only in the *in vitro* treatment of the implantation site explants with VIP but also in the *in vivo* treatment of pregnant mice at the early post implantation period. On the one hand, VIP decreased the Th17 response reflected by a reduction in IL-17 and RORγ expression after *in vitro* and *in vivo* treatments. This effect was accompained by an increase in the expression of immuno suppressor modulators as TGF-β and PPARγ at the implantation sites. On the other hand, VIP modulated the efferocytosis of macrophages evaluated by two complementary approaches, the efferocytosis of latex beads and of apoptotic thymocytes. In addition, VIP treatment *in vivo* increased the frequency of F4/80+ IL-10-producing cells suggesting their activation in an alternative profile.

VIP immunomodulatory effect has been observed in the NOD mice, evidenced by the induction IL-10, TGF-β and Foxp3 expression at the implantation sites. In addition, implantation sites undergoing resorption processes showed lower VIP expression along with a lower expression of these cytokines and the transcription factor Foxp3, higher prostaglandin synthesis and higher expression of IL-17 and RORγt^12^,^30^.

Recently, Obermajer *et al.* demonstrated the conversion of Th17 cells into Treg by which mesenchymal stem cells induced myeloid-derived immunosuppressive cells that mediate operational transplant tolerance. They demonstrated that RORγ is a common factor in the differentiation of Treg and Th17 cells in mice, and the identification of IL-17A + Foxp3+ cells that then converts into a IL-17A-Foxp3+ cell strongly argues for direct conversion of Th17 cells into Treg cells^31^. Here, in the CBAxDBA model, we show that VIP treatment decreased RORγ and slightly increase Foxp3 expression. However, whether this new mechanism of immune regulation operates at the feto-maternal interface is still an open question.

With respect to the distribution of intrauterine embryo implantation sites, in most mammalian species shows remarkably constant patterns. In rodents, embryos implant evenly along the uterine horns and the disruption of these patterns can have adverse effects on pregnancies. Chen Q. *et al.*, describe diverse molecular factors, such as steroid hormone signaling, lipid signaling, adrenergic signaling, developmental genes, ion/water channels, and potentially embryonic signaling are actively involved in intrauterine embryo distribution^32^; even VIP treatment improve symmetric distribution it will be interesting to investigate VIP efects on this potential mediators.

Regarding macrophage phenotypes, the classification is useful but represents an over simplification due to their substantial plasticity programming during the course of an inflammatory response, with markers and functions readily altered by external signals.

The activation of macrophages in an alternative profile has been associated with enhanced efferocytosis of apoptotic cells preventing the release of intracellular contents that can contribute to inflammation and autoimmunity^33^. Collectively, these macrophages exhibit increased expression and/or activity of the nuclear receptors, PPARγ and PPARδ, essential to their acquisition of alternative activation profile, associated with increased levels of arginase, certain receptors and anti-inflammatory cytokines^27^,^28^.

Here we observed that *in vivo* VIP treatment improves efferocytosis evaluated by two complementary approaches, the use of latex beads and apoptotic thymocytes. Moreover, we also observed an increased expression of TGF-β and PPARγ at the implantation sites.

In this sense, in a murine model of chronic granulomatous disease (CGD), which exhibits chronic inflammation and mild autoimmunity, macrophages maintain a pro-inflammatory profile associated with an impaired efferocytic ability and decreased expression of PPARγ^34^,^35^. In fact, the treatment with a PPARγ agonist in this mouse model of CGD, resolved prolonged zymosan-induced inflammation reflected by enhanced efferocytosis, and increased TGF-β and IL-10 production, highlighting a direct association between PPARγ increased expression and improved efferocytosis^34^. The macrophage PPARγ activation, increased efferocytic surface receptors and secretion of the bridge molecules, then enhanced efferocytic ability and the suppression of inflammation^36^.

These results also agree with previous data showing that VIP contributes to the switch of peritoneal macrophage predominant profile from an ‘inflammatory’ to a ‘regulatory’ phenotype at early pregnancy stages with increased production of IL-10^37^.

The cellular mechanisms that participate in VIP effect may involve multiple cells among which decidual cells and macrophages targeted by the peptide could contribute to a suitable immune tolerant microenvironment. Here, we show that the *in vivo* shift from inflammation to a pro-resolving state could be attributed, at least in part, to macrophages switching their programming in response to the changing milieu, suggesting that this peptide might be crucial to local homeostasis control at feto-maternal interface immunosuppressant mediators for adequate fetal growth.

## Methods

### Mice matings and treatment

An immunological model of exacerbated inflammation was represented by the mating combination CBA/J × DBA/2 and the combination CBA/J x BALB/c was used as a normal allogenic pregnancy (control mating). Mice were bred and maintained on a 12:12 h light–dark schedule in the Central Animal Care facility at the School of Exact and Natural Sciences, University of Buenos Aires (FCEyN-UBA).

Two-month-old CBA/J females were paired with BALB/c or DBA/2 males, checked daily for vaginal plugs and separated once mated. The day of the vaginal plug was considered as day 0.5 of pregnancy. The animals included in the first part of the study received no treatment. In the second part of the study, pregnant CBA/J females mated with DBA/2 or BALB/c were treated with phosphate-buffered saline (PBS) intraperitoneally or with VIP (2 nmol/mouse) at day 6.5 of gestation. Pregnant females were killed at day 8.5 of gestation. All studies were conducted according to standard protocols and were approved by the Animal Care and Use Committee of the School of Sciencies, University of Buenos Aires. An asymmetry function was used to assess the effect of VIP on the distribution of impanted embryos along the horns, taking into account the number of embryo and the distance in each horn.

### Uterus and implantation site isolation for functional studies

Pregnant uteri were carefully dissected at day 8.5 of pregnancy and implantation sites were defined as previously described^37^ and isolated for cytokine and transcription factors measurement^23^. Explants of implantation sites (inlcuded placenta, decidua and mesometrial lymphoied aggregate of pregnacy, MLAp tissue) were excised, embryos discarded and tissues were incubated at 37 °C and 5%CO_2_ with VIP (100 nM) (Polypeptide labs, France) or not. Sites with viable embryos and sites with incipient resorption processes at gestational day 8.5 were assessed under magnifying glass, quantified to calculate resorption rate and photographed to show embryo distribution along horns among other gestation outcome signs as previously described^12^,^23^.

### VIP/VPAC, Foxp3, RORγt and pro/anti inflammatory mediator detection

The expression of VIP and VPAC receptors, transcription factors and pro/anti inflammatory mediators was determined by RT-PCR. Briefly, total RNA was isolated following manufacturer recommendations with Trizol reagent (Life Technologies, Grand Island, NY, USA), cDNAs were generated from 1 ug or RNA using a MMLV reverse transcriptase, RNAsin RNAse inhibitor and oligodT kit (Promega Corporation, Madison, WI, USA) and stored at −20 °C for batch analysis. The sample volume was increased to 25 ul with the solution containing 50 mM KCl; 10 mM Tris (pH 8.3); 1.5 mM MgCl2; 0.1 mM forward and reverse primers of VIP, VPAC_1_, VPAC_2_, TGF-β, IL-17, PPRγ, Foxp3, RORγT and GAPDH as internal control (described in Table 1) and 1 U Taq polymerase in a DNA Thermocycler (PerkinElmer/Cetus, Boston, MA, USA). PCR products were electrophoresed through a 2% ethidium bromide-stained agarose gel, visualized by transillumination and scanned. Densitometry was performed using ImageJ 1.47 software (NIH, USA) and results expressed as arbitrary units normalized to GAPDH expression. Each assay included a DNA minus control and a standard curve performed with serial dilutions of control cDNA.

### Peritoneal macrophages isolation

Macrophages were obtained from pregnant CBA/J × DBA/2 mice at day 8.5, treated with PBS or VIP at day 6.5. The peritoneal cavity was washed with ice-cold HANKS as reported^38^ were resuspended in RPMI 10% fetal calf serum (FCS) (Natocor Life Technologies, MD) and seeded in 24-well plates (Corning Glass, Corning, NY) at 5 × 10^5^ cells/well. After incubation at 37 °C for 2 h, monolayers (>95% macrophages by F4/80 flow cytometry staining) were used for phagocytosis assays.

### Efferocytosis assays

To evaluate the efferocytosis ability we used two complementary approaches, the phagocytosis of latex beads-FITC and of apoptotic thymocytes.

*Efferocytosis of latex beads-FITC.* Peritoneal macrophages recovered from CBA/J × DBA/2 female at 8.5 day of gestation were cultured with latex beads (1–3 μm) FITC-conjugated in the absence or presence of VIP (10 nM) in a 1:100 ratio (macrophage:beads). Incubations were performed at 37 °C with 5% CO_2_ in 24-well plates and after 90 min macrophages were recovered by Triple® (Gybco, Invitrogen, Argentina) and the percentage of efferocytosis was quantified by FACS analysis. Results are expressed as % of FITC+ cells.

*Efferocytosis of apoptotic thymocytes.* Thymocytes were obtained from syngeneic mice of 21 days of age, were thoroughly washed and induced to apoptosis with 100 nM dexamethasone for 4 h at 37 °C^38^. Then macrophages were co-cultured with syngeneic thymocytes previously induced to apoptosis (ratio 1:5 macrophage: thymocytes). Incubations were run at 37 °C on coverslips placed inside 24-well plates for the indicated times (30, 60, 90 and 120 min), then stained with Hematoxilin–Eosin and analyzed by microscopy. Results are expressed as the % of efferocytosis and the phagocytic index determination representing the number of apoptotic bodies phagocytized by each macrophage^38^.

### Cytokine quantification

After the efferocytosis assay, macrophages from pregnant CBA/J females treated with PBS or VIP were washed and remained in culture for additional 24 h and then supernatants were collected for cytokine determination. Cytokines were also evaluated before the efferocytosis assay in both groups of mice under study. ELISA for TNF-α and IL-12 (eBiosciences, USA) were performed according to the manufacturer’s protocols. The limits of detection were 8 pg/ml for TNF-α and 4 pg/ml for IL-12. Results were expressed in pg/ml.

### Flow-cytometric analysis

#### Intracellular staining for IL-10 detection

IL-10 production was quantified in macrophages of pregnant CBA/J females after *in vivo* treatment with PBS or VIP before and after efferocytosis. In the last 4 h of the assay, Stop Golgi (BD, Pharmigen, San José, CA, USA) was added to the culture to promote IL-10 intracellular accumulation. Then macrophages were recovered and after washing, cells were stained with anti-F4/80-FITC mAb for 30 min, fixed with Cytofix/cytoperm (BD, Pharmigen, San José, CA, USA) at room temperature and incubated for 30 min with anti-IL10-PE mAb (eBiosciece, USA).

Ten thousand events were acquired in a FACSAria cytometer^®^ and results were analyzed using the FlowJo software^®^. Negative control samples were incubated in parallel with an irrelevant isotype-matched antibody. Results for positive cells are expressed as a percentage of F4/80 + IL-10+ cells.

### Statistical analysis

Statistical significance of differences was determined by the two-tailed t test for independent populations and Student’s *t* test for parametric analysis. When multiple comparisons were necessary, the Student-Newman-Keuls test was used after analysis of variance. For *in-vivo* treatment experiments, the Mann-Whitney Test was used. Statistical significance was defined as p < 0.05, using the GraphPad Prism6 software (GraphPad, San Diego, CA, USA).

## Additional Information

**How to cite this article**: Gallino, L. *et al.* VIP treatment prevents embryo resorption by modulating efferocytosis and activation profile of maternal macrophages in the CBAxDBA resorption prone model. *Sci. Rep.* **6**, 18633; doi: 10.1038/srep18633 (2016).

## Figures and Tables

**Table 1:** *Primers used in RT-PCR assays.*

*Primers*	Forward	Reverse
GAPDH	TGATGACATCAAGAAGGTGGTGAAG	TCCTTGGAGGCCATGTAGGCCAT
VIP	TTCACCAGCGATTACAGCAG	TCACAGCCATTTGCTTTCTG
VPAC_1_	GTGAAGACCGGCTACACCAT	TGAAGAGGGCCATATCCTTG
VPAC_2_	GTGAAGACCGGCTACACCAT	TGAAGAGGGCCATATCCTTG
PPARγ	ATC TAC ACG ATG CTG GC	GGA TGT CCT CGA TGG
TGF-β	GACTCTCCACCTGCAAGACCA	TTGGGGGACTGGCGAGCCTT
Foxp3	GGCCCTTCTCCAGGACAGA	GCTGATCATGGCTGGGTTGT
IL-17	CTCCAGAAGGCCCTCAGACTAC	AGCTTTCCCCTCCGCATTGACACAG
RORγt	CACGGCCCTGGTTCTCAT	CAGATGTTCCACTCTCCTCTTCTCT

Oligonucleotide primers were designed using the online tool Primer3® (Whitehead Institute for Biomedical Research).
